# Mechanistic diversity and functional roles define the substrate specificity and ligand binding of bacterial PGP phosphatases

**DOI:** 10.1016/j.jbc.2024.107959

**Published:** 2024-11-05

**Authors:** Wei Niu, Joanne Shi Woon Lam, Trung Vu, Guangwei Du, Hao Fan, Lei Zheng

**Affiliations:** 1 Department of Biochemistry and Molecular Biology, Center for Membrane Biology, University of Texas Health Science Center at Houston McGovern Medical School, Houston, Texas, USA; 2 Bioinformatics Institute (BII), Agency for Science, Technology and Research (A∗STAR), Singapore, Republic of Singapore; 3 Department of Integrative Biology and Pharmacology, University of Texas Health Science Center at Houston McGovern Medical School, Houston, Texas, USA; 4 Synthetic Biology Translational Research Program, Yong Loo Lin School of Medicine, National University of Singapore, Singapore, Republic of Singapore; 5 DUKE-NUS Medical School, Singapore, Republic of Singapore

**Keywords:** phospholipids, phosphatase, membrane, phosphatidylglycerol, ligand binding

## Abstract

Phosphatidylglycerol is a critical membrane phospholipid in microorganisms, synthesized *via* the dephosphorylation of phosphatidylglycerol-phosphate (PGP) by three membrane-bound phosphatases: PgpA, PgpB, and PgpC. While any one of these enzymes can produce phosphatidylglycerol at WT levels, the reason for the presence of all three in bacteria remains unclear. To address this question, we characterized these phosphatases *in vitro* to uncover their mechanistic differences. Our assays demonstrated that all three enzymes catalyze the hydrolysis of PGP but exhibit distinct substrate selectivity. PgpB displays a broad substrate range, dephosphorylating various lipid phosphates, while PgpA and PgpC show a higher specificity for lysophosphatidic acid and PGP. Notably, PgpA also effectively dephosphorylates soluble metabolites, such as glycerol-3-phosphate and glyceraldehyde-3-phosphate, suggesting its unique substrate-binding mechanism that relies on precise recognition of the glycerol head group rather than the fatty acid. Inhibitor screening with synthetic substrate analogs revealed that PgpB is inhibited by lipid-like compounds XY-14 and XY-55, whereas PgpA and PgpC are unaffected. Structural analysis and mutational studies identified two charged residues at the catalytic site entry for inhibitor binding in PgpB and support the notion that the PgpB maintains a large substrate binding site to accommodate multiple ligand binding conformations. These findings underscore the distinct substrate recognition mechanisms and possible functional roles of PgpA, PgpB, and PgpC in bacterial lipid metabolism and offer insights for developing novel inhibitors targeting bacterial membrane phospholipid biosynthesis.

Phosphatidylglycerol (PG) is an abundant anionic phospholipid present in nearly all prokaryotic cell membranes (1, 2). It constitutes the major phospholipid in gram-positive bacteria and makes up to 20% of the total phospholipid composition in *Escherichia coli*. PG is crucial for bacterial growth due to its role in maintaining membrane structure and function. For instance, it stabilizes cell membranes by promoting a lateral bilayer conformation (3) and regulates membrane protein functions and various biological processes, including DNA replication initiation at oriC and SecA-dependent protein translocation (4, 5). Additionally, PG is a key metabolic intermediate, serving as a precursor for several important cellular metabolites. For instance, it condenses with another PG molecule or phosphatidylethanolamine (PE) to form cardiolipin, an essential lipid for bacterial respiration and cell division (6). PG also contributes to the biosynthesis of outer membrane lipoproteins and membrane-derived oligosaccharides (7, 8). In gram-positive bacteria, it is often modified with amino acids to form lysyl-, alanyl-, or arginyl-PG, constituting up to 40% of the total lipid composition (9, 10). Although PG is a common lipid in many bacteria, it is worth noting that some bacteria, such as those in the genus *Mycobacterium* and certain species of Corynebacterium, do not contain PG in their membranes (11, 12).

In prokaryotes, PG and other phospholipids are synthesized through a conserved metabolic pathway, the so-called Kennedy pathway (1, 2, 13, 14) (Fig. 1). This pathway begins with lysophosphatidic acid (LPA), which is acylated to form phosphatidic acid (PA). PA then reacts with CTP to produce CDP-diacylglycerol (CDP-DAG), a key intermediate for phospholipid biosynthesis. PG is ultimately formed from CDP-DAG through a two-step process: first, the CMP moiety of CDP-DAG is replaced by glycerol-3-phosphate (G3P) to form phosphatidylglycerol-phosphate (PGP); second, PGP is dephosphorylated to generate PG.

**Figure 1:**
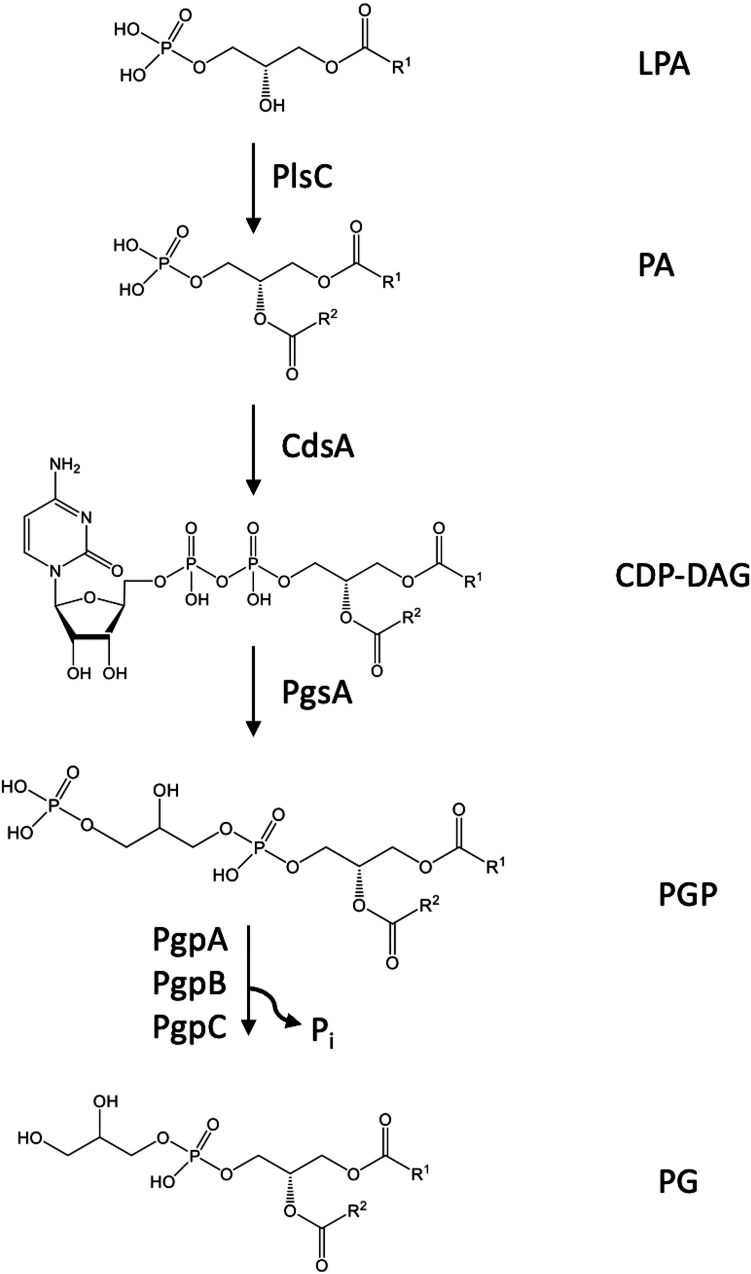
**Schematic representation of phosphatidylglycerol biosynthesis pathway in *Escherichia coli* inner membrane.** Phosphatidylglycerol (PG) is synthesized through sequential reactions in the bacterial inner membrane, starting with lysophosphatidic acid (LPA) as the precursor. The enzyme 1-acyl-sn-glycerol-3-phosphate acyltransferase PlsC catalyzes the transfer of an acyl group from LPA to produce phosphatidic acid (PA). Subsequently, phosphatidate cytidylyltransferase CdsA transfers a cytidyl group from CTP to PA, forming CDP-diacylglycerol (CDP-DAG). CDP-DAG glycerol-3-phosphate 3-phosphatidyltransferase PgsA then substitutes the CMP moiety of CDP-DAG with glycerol-3-phosphate to generate phosphatidylglycerol-phosphate (PGP). In the final step, three PGP phosphatases—PgpA, PgpB, and PgpC—catalyze the dephosphorylation of PGP to produce PG. For clarity, the fatty acid chain at the sn-1 or sn-2 position of lipids is abbreviated as R1 or R2. PA, phosphatidic acid.

In *E. coli* and many other bacteria, the dephosphorylation of PGP is carried out by three distinct membrane phosphatases: PgpA, PgpB, and PgpC (15, 16, 17). Despite performing the same reaction, these enzymes are unrelated in sequence and membrane topology. PgpA and PgpB are multispanning transmembrane proteins, whereas PgpC is predicted to be a peripheral protein associated with the cytoplasmic membrane surface (15, 18). Additionally, the enzymes appear to use different catalytic mechanisms: PgpA and PgpC require Mg^2+^ for activity, while PgpB does not (15). These varying Mg^2^⁺ dependencies are consistent with the predicted orientations of their active sites on different sides of the inner membrane since millimolar concentrations of Mg^2^⁺ are typically found in the cytoplasm rather than in the periplasm. The triple knockout of *pgpA*, *pgpB*, and *pgpC* genes is lethal in *E. coli*. However, the redundancy of these phosphatases is puzzling, as the deletion of any two of them does not affect PG levels, suggesting that a single phosphatase is sufficient for PG production. One hypothesis is that these enzymes have additional functions beyond PGP dephosphorylation. For instance, PgpB is also involved in peptidoglycan biosynthesis by dephosphorylating undecaprenyl pyrophosphate (C55-PP) (19). Although PgpA and PgpC have not been shown to participate in other cellular processes, mutations in their genes have been associated with reduced bacterial tolerance to high temperatures, indicating potential roles in stress responses (15). To date, the structure and functional studies of these PG-producing enzymes are only limited to PgpB (18, 19, 20) with no *in vitro* characterizations reported for PgpA and PgpC.

To elucidate the functional differences among these phosphatases, we purified PgpA, PgpB, and PgpC from *E. coli* and analyzed their substrate selectivity *in vitro*. Our findings reveal the distinct substrate profiles of these three enzymes, reflecting their unique substrate-binding mechanisms. We also identified specific inhibitors of PgpB through synthetic substrate analog screening and characterized the binding mechanisms using ligand docking and mutational analysis. These studies indicate that PgpB and PgpC may primarily act on lipid ligands with distinct specificity, whereas PgpA displays unique activity with smaller phosphorylated metabolites. These results offer insights into the distinct functions of the three phosphatases in lipid biosynthesis and may inform the development of novel inhibitors targeting bacterial phospholipid biosynthesis.

## Results

### Distinct selectivity for lipid phosphate substrates

**Figure 2:**
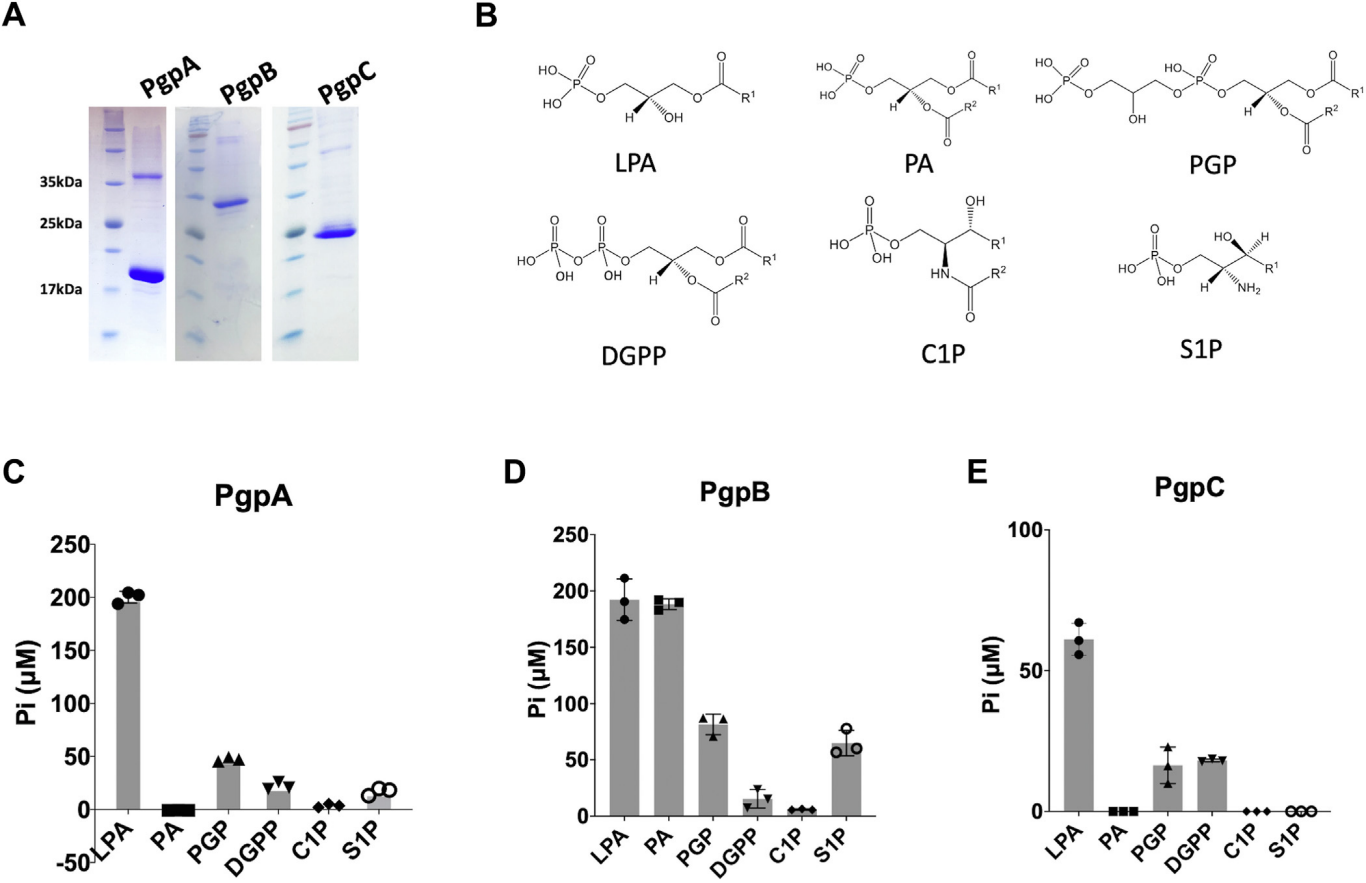
**Distinct lipid substrate selectivity of PgpA, PgpB, and PgpC.***A,* coomassie-stained SDS-PAGE gel images showing His-tagged PgpA, PgpB, and PgpC proteins from *Escherichia coli* purified using Ni-NTA resins. *B,* chemical structures of the lipid phosphate substrates used in this study: 18:1 lysophosphatidic acid (LPA), 16:0 phosphatidic acid (PA), 18:1 phosphatidylglycerol-3-phosphate (PGP), 16:0 ceramide-1-phosphate (C1P), and 16:0 sphingosine-1-phosphate (S1P). For clarity, the fatty acid chain at the sn-1 or sn-2 position of lipids is abbreviated as R1 or R2. *C–E, in vitro* phosphatase activities of PgpA (*C*), PgpB (*D*), and PgpC (*E*) measured by detecting inorganic phosphate (Pi) release using the colorimetric phosphate assay. Reactions (100 μl) containing 0.5 μg PgpA, 0.05 μg PgpB, or 1.5 μg PgpC protein and 10 μg of each substrate were incubated at room temperature for 30 min. Activities were normalized against individual background controls. All data are provided as scatter dot plots superimposed with mean and SD, n = 3.

To explore the functionality of PGP phosphatases *in vitro*, we expressed and purified the *E. coli* PgpA, PgpB, and PgpC proteins, each tagged with an N-terminal His tag, in a nonionic detergent solution (Fig. 2*A*). We measured lipid phosphatase activities by detecting inorganic phosphate (Pi) release using a malachite green phosphate assay. In the assay, Pi reacts with malachite green dye to form a complex, which can be quantified spectroscopically. Initial assays demonstrated robust dephosphorylation of PGP by all three phosphatases, confirming their active state (Fig. 2, *C–E*). We then assessed their substrate selectivity by evaluating their activities against a range of lipid phosphate analogs, including LPA, PA, diacylglycerol pyrophosphate (DGPP), sphingosine-1-phosphate (S1P), and ceramide-1-phosphate (C1P) (Fig. 2*B*). The rationale for including these substrates was as follows: i) LPA and PA are two crucial precursors for PG and other phospholipids (Fig. 1); ii) DGPP, a closed structural analog of PG with a pyrophosphate head group, is present in bacteria, plants, and mammals, and its dephosphorylation has been previously observed in *E. coli* (21, 22); iii) S1P and C1P, structural analogs of LPA or PA, contain an amino-hydroxyl group in place of the glycerol backbone found in LPA/PA/PGP (Fig. 2*B*).

Our results indicate that LPA is the preferred substrate for all three enzymes, with PgpA, PgpB, and PgpC showing over 3-fold higher activity with LPA than PGP (Fig. 2, *C–E*). However, testing with other lipid phosphate substrates reveals distinct selectivity profiles for each phosphatase. PgpB hydrolyzed both LPA and PA with equal efficiency, while PgpA and PgpC showed no activity toward PA, likely due to steric hindrance from the sn-2 acyl group. PgpA and PgpC also exhibited lower activity with DGPP than PGP, suggesting that the elongated pyrophosphate headgroup partially alleviates the steric restriction. Additionally, the substitution of the glycerol backbone with other groups, as seen in S1P and C1P, significantly reduced the activity of PgpA and PgpB, with no activity observed for PgpC. These findings imply that the glycerol backbone is crucial for substrate recognition, particularly for PgpA and PgpC.

**Figure 3:**
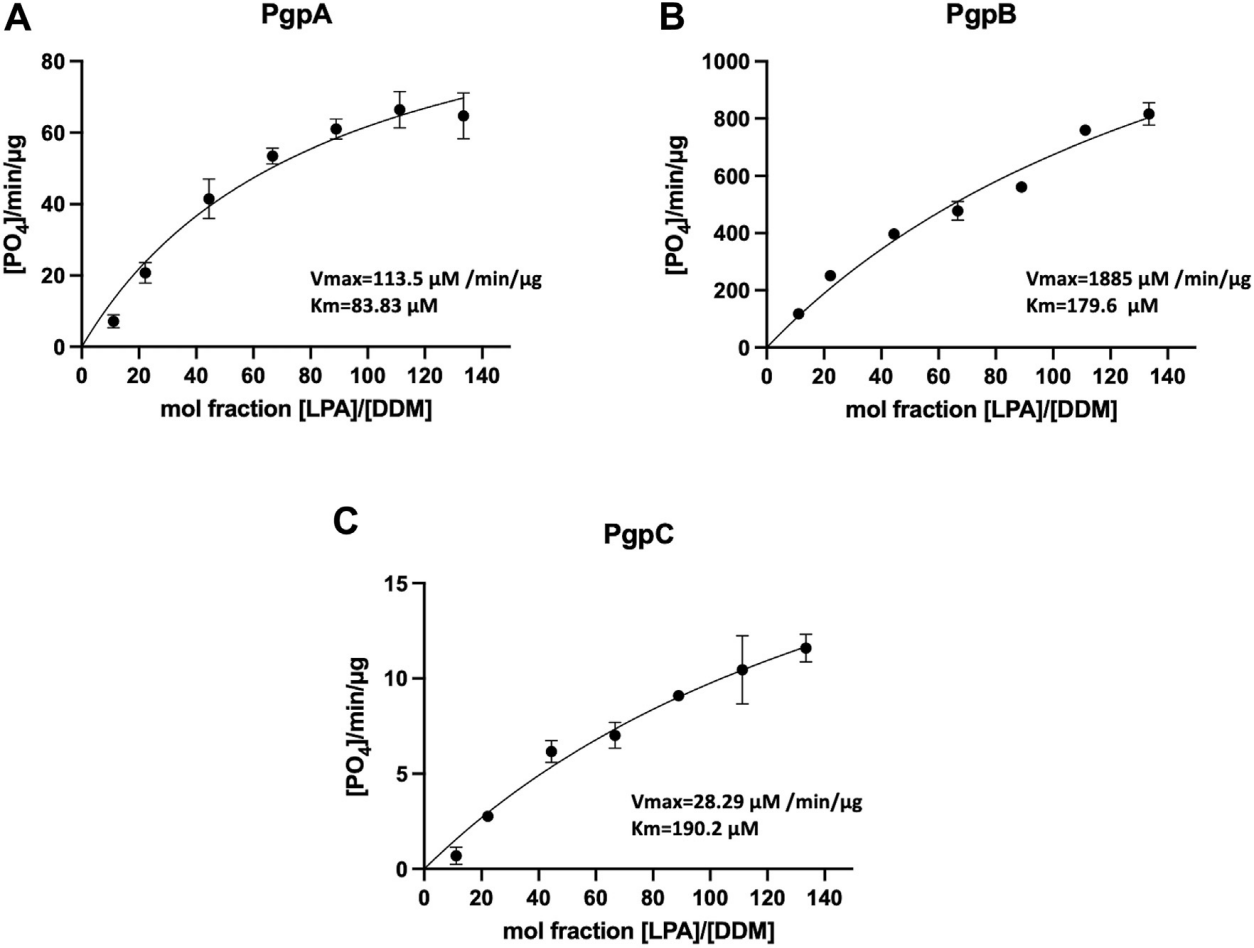
**Kinetics of lipid phosphatase activity of PgpA, PgpB, and PgpC.** Kinetic analyses of PgpA (*A*), PgpB (*B*), and PgpC (*C*) using 18:1 lysophosphatidic acid (LPA) as the substrate. Reactions (100 μl) containing appropriate amounts of PgpA, PgpB, or PgpC protein and different concentrations of LPA: 0.109, 0.218, 0.436, 0.654, 0.872, 1.090, and 1.308 mM were incubated at room temperature for 5 min. Molar fractions were calculated based on 0.98 mM DDM in the reactions. Data fitting was performed using the surface dilution model with the GraphPad Prism software. Error bars represent SDs (n = 3).

We further characterized the enzyme kinetics using LPA, given its high activity with all three enzymes. Kinetic parameters were determined by fitting the data to a surface dilution model (Fig. 3, *A–C*). PgpA displayed a slightly higher substrate binding affinity (*K*_*m*_ = 83.8 μM) than PgpB (*K*_*m*_ = 179.6 μM) and PgpC (*K*_*m*_ = 190.2 μM). However, PgpB exhibited a significantly higher catalytic activity (*V*_*max*_ = 1885 μM/min/μg) than PgpA (*V*_*max*_ = 113.5 μM/min/μg) and PgpC (*V*_*max*_ = 28.3 μM/min/μg). These results suggest that PgpB has the highest catalytic capacity and a broader substrate range, whereas PgpA and PgpC are more specific for PGP and LPA.

### Unique catalysis of PgpA with soluble metabolites

PgpA, PgpB, and PgpC are membrane-embedded enzymes thought to perform lipid metabolism in the lipid bilayer. The structure of PgpB suggests that the lipid dephosphorylation reaction occurs on the membrane surface, with the active site exposed to the periplasmic space and primarily assembled by several charged residues (18, 20). Although the structures of PgpA and PgpC are not yet available, the AlphaFold-predicted models suggest that their active sites may also be located on the cytoplasmic membrane surface (23). This surface localization may enable these PGP phosphatases to act on other soluble phosphate metabolites.

**Figure 4:**
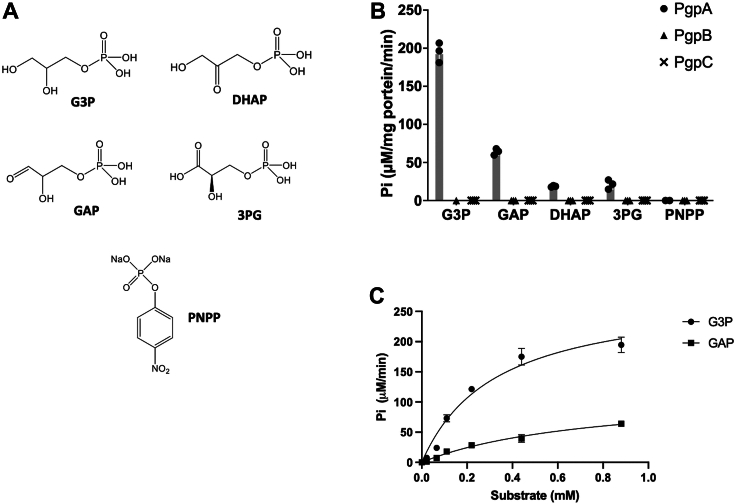
**Unique phosphatase activity of PgpA with 3-carbon sugar phosphate metabolites.***A,* chemical structures of soluble phosphate metabolites and substrate used in this study: glycerol-3-phosphate (G3P), dihydroxyacetone phosphate (DHAP), DL-glyceraldehyde-3-phosphate (GAP), D-3-phosphoglyceric acid (3PG), and p-nitrophenyl phosphate (PNPP). *B,* phosphatase activities of PgpA, PgpB, and PgpC measured *in vitro* by detecting inorganic phosphate (Pi) release using the colorimetric phosphate assay. Reactions (100 μl) containing 2 μg of proteins and 5 μg of each substrate were incubated at room temperature for 40 min. Activities were normalized with an individual background control. All data are provided as scatter dot plots superimposed with mean and SDs (n = 3). *C,* kinetic assays of PgpA measured with G3P and GAP. The activity was normalized against individual background controls. Data fitting to the Michaelis–Menten kinetic model was performed using GraphPad Prism.

To test this hypothesis, we focused on four 3-carbon sugar phosphate metabolites, including G3P, glyceraldehyde-3-phosphate (GAP), dihydroxyacetone phosphate (DHAP), and 3-phosphoglyceric acid (3PG)—which are structurally analogous to the PG head group of LPA (Fig. 4*A*). These metabolites are crucial building blocks for cellular metabolism (24). For instance, G3P, generated in the glycerol metabolic pathway, can be directly acylated to form LPA and is also used by PgsA to synthesize PGP (Fig. 1); GAP, DHAP, and 3PG are metabolic intermediates in the glycolytic pathway, with GAP and DHAP produced by the breakdown 1,6-bisphosphate fructose and subsequently converted to 3PG before entering the TCA cycle.

We tested PgpA, PgpB, and PgpC with these compounds (Fig. 4*B*). Our results showed that neither PgpB nor PgpC acted on these soluble phosphate metabolites, despite PgpB’s broad lipid substrate selectivity. In contrast, PgpA exhibited strong Mg^2+^-dependent dephosphorylation activity with G3P. The PgpA activity was also strongly dependent on the conformation of these 3-carbon sugars, that is, it was significantly reduced (4-fold) with GAP and was nearly absent with 3PG. Notably, the only difference between these molecules is at the sn-2 position, where GAP or 3PG has a carbonyl oxygen or carboxylate group instead of a hydroxyl group present in G3P. A similar effect was observed with DHAP, where a carbonyl oxygen group at the sn-2 position disrupts the activity. These data suggest that PgpA employs a highly specific substrate-binding mechanism that recognizes the glycerol group. Kinetic assays further confirmed these findings, with *K*_*m*_ and *V*_*max*_ values for G3P (*K*_*m*_ = 0.327 mM, *V*_*max*_ = 281.3 μM/min/μg) being significantly better compared to GAP (*K*_*m*_ = 0.765 mM, *V*_*max*_ = 117.8 μM/min/μg) (Fig. 4*C*). No activity was observed with p-nitrophenyl phosphate, a standard phosphatase substrate, underscoring specific binding of G3P and GAP. (Fig. 4*B*). PgpB and PgpC did not exhibit activity towards these soluble metabolites, highlighting PgpA's unique substrate recognition and its potential role in regulating bacterial cellular metabolisms.

### Identification of PgpB inhibitors

**Figure 5:**
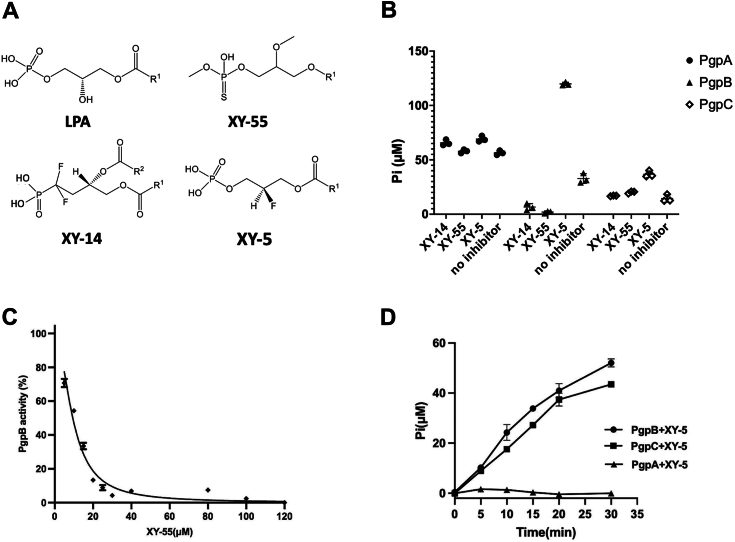
**Identification and characterization of PgpB inhibitors.***A,* chemical structures of lipid-like synthetic compounds, XY-14, XY-55, XY-5 in comparison to lysophosphatidic acid (LPA). For clarity, the fatty acid chain at the sn-1 or sn-2 position of lipids is abbreviated as R1 or R2. *B,* phosphatase activities of PgpA, PgpB, and PgpC measured in the presence of 65 μM XY-14, 107 μM XY-55, or 115 μM XY-5 using the colorimetric phosphate assay. Reactions (100 μl) containing 0.5 μg PgpA, 0.05 μg PgpB, or 1.5 μg PgpC protein and 5 μg LPA were incubated at room temperature for 5 min. Activities were normalized against individual background controls. All data are provided as scatter dot plots superimposed with mean ± standard deviation (n = 3). *C,* kinetic assays of XY-55 inhibition. PgpB was preincubated with varying concentrations (5–100 μM) of XY-55 for 10 min before adding 5 μg LPA to trigger the reaction, which was carried out for 5 min. Data was fitted to the single-exponential binding model using GraphPad Prism. *D,* phosphatase activity of PgpA, PgpB, or PgpC measured using 5 μg XY-5 in 100 μl reactions. Activities were normalized against individual background controls. Data (n = 3) are given as mean ± standard deviation.

PGP dephosphorylation is a crucial lipid metabolic reaction essential for bacterial survival as the simultaneous knockdown of *pgpA, pgpB, and pgpC* genes is lethal to *E. coli* (15). To date, no specific inhibitors targeting this essential bacterial catalysis have been reported. Given that LPA is the preferred substrate for PgpA, PgpB, and PgpC (Fig. 2, *C–E*), we hypothesized that structural analogs of LPA could serve as universal inhibitors for all three enzymes. We then tested several metabolically stabilized LPA analogs reported by Prestwich and colleagues for studying mammalian LPA receptors or lipid phosphatases (25, 26, 27). None of these analogs inhibited all three enzymes simultaneously. However, two compounds, XY-14 and XY-55, were found to selectively inhibit PgpB, with no effect on PgpA or PgpC. XY-14 is a diacyl PA structural analog with two fluorine substitutions at the sn-1 position (Fig. 5*A*). As shown in Figure 5*B*, adding 65 μM XY-14 reduced PgpB activity by >90%. Notably, XY-14 was previously found as an inhibitor of lipid phosphate phosphatase, a PgpB homolog in humans (28), suggesting a conserved ligand-binding mechanism within the lipid phosphate phosphatase family. Different from XY-14, XY-55 is a thiol-phosphate LPA analog, and it also has a methoxy group at its sn-2 position (Fig. 5*A*). As shown in Figure 5*B*, XY-55 completely inhibited PgpB at ∼100 μM. We further characterized XY-55 by measuring its inhibitory potency (IC_50_). The data was fitted to a single-exponential binding model, yielding an IC_50_ value of 17.24 μM (Fig. 5*C*). To date, no reports have indicated that XY-55 inhibits mammalian LPP homologs, making a potential specific inhibitor of bacterial PgpB.

Among screened LPA analog compounds, XY-5 is distinguished by having fluorine at the sn-2 position instead of a hydroxyl group (Fig. 5*A*). We discovered that XY-5 acts as a substrate for PgpB/PgpC, not for PgpA. Specifically, the addition of XY-5 in the LPA reactions of PgpB and PgpC resulted in a more than 3-fold increase in Pi release (Fig. 5*B*). In contrast, no such effect was observed with PgpA. Further experiments with XY-5 alone (without LPA substrate) revealed that only PgpB and PgpC, not PgpA, could dephosphorylate XY-5 as evidenced by the release of Pi into the solutions over time (Fig. 5*D*). This observation aligns with soluble metabolite assays, suggesting that the sn-2 hydroxyl group may be crucial for PgpA substrate recognition.

### Hypothetic mechanism of inhibitor binding

The identification of the XY-55 inhibitor provides an opportunity to explore the ligand-binding mechanism of PG metabolic enzymes. To gain structural insight into XY-55 binding, we implemented *in silico* ligand docking using the crystal structure of PgpB previously determined by our group (18). Ligand docking of LPA and XY-55 was conducted using a similar approach to allow for parallel comparison of substrate and inhibitor binding. The final docking poses were selected based on the best docking scores, which are similar for LPA (−9.272) and XY-55 (−8.621).

**Figure 6:**
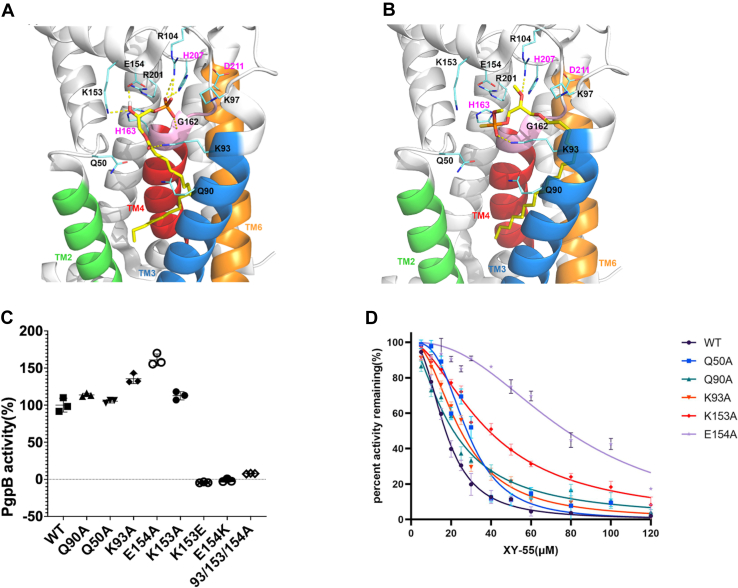
**Hypothetic mechanism of PgpB inhibitor XY-55.***A* and *B,* docking conformations of lysophosphatidic acid (LPA) (*A*) and XY-55 (*B*) at the catalytic site of PgpB. PgpB [PDB ID: 5JWY] is shown as a ribbon schematic; transmembrane helices TM2 (*green*), TM3 (*blue*), PSGH loop (*pink*), TM4 (red), TM6 (*orange*), key residue side chains (*cyan blue*), and ligands (*yellow sticks*). Binding conformations of a) LPA and b) XY-55 depict their phosphate head groups located deeply within the active site and proximal to or interacting with residues of the catalytic triad/phosphate binding site. XY-55 shows that their headgroups adopt a different orientation in PgpB active site from that of LPA and XY-5 with head groups facing away from the active site. *C,* LPA activity of PgpB mutants expressed as a percentage compared to WT. Reactions (100 μl) containing 50 ng protein and 5 μg of LPA were incubated at room temperature for 5 min. All data points are provided as scatter dot plots with error bars representing mean and SD (n = 3). *D,* XY-55 binding (IC_50_) to various PgpB mutants. LPA activities at different XY-55 concentrations were fitted to single potential binding curves. Compared with the WT (IC_50_ = 17.24 μM), the IC_50_ values of XY-55 to K153A (IC_50_ = 38.94 μM) and E154A (IC_50_ = 77.71 μM) are increased. Error bars represent SDs (n = 3). TM3, transmembrane helix 3.

In PgpB, its lipid binding pocket includes an active site and a membrane-embedded lipid tunnel. The active site contains a phosphate-binding site (Lys97, Arg104, Arg201) and a catalytic triad (His163, His207, and Asp211). The lipid tunnel is formed by the transmembrane helix 3 containing a lysine residue Lys93, and PSGH (Pro160, Ser161, Gly162, His163) fingerprint peptide located in TM4 (Fig. 6*A*). Docking simulations show that the phosphate headgroup of LPA forms multiple hydrogen bonds with His207 and Arg104 side chains and Gly162 backbone. LPA binding is further stabilized by hydrogen bonding between the LPA hydroxyl moiety and Lys153/Glu154 side chains, as well as interactions between the LPA carbonyl moiety and Lys93 side chain, and hydrophobic interactions between the LPA acyl tail and surrounding lipid tunnel residues, including Leu82 and Ile86 in transmembrane helix 3 and Phe166 and Trp170 in TM4. This docking pose suggests that LPA binds to the PgpB active site in a manner that facilitates its dephosphorylation.

In contrast, while XY-55 adopts a similar orientation in the lipid tunnel as LPA (Fig. 6*B*), its head group faces away from the catalytic triad in the active site, exhibiting a significantly different orientation from LPA. XY-55 forms hydrogen bonds between its phosphorothioate head group and the Lys93 side chain amine group and between its acyl tail methoxy moiety and Arg104 side chain guanidinium group.

To test this model, we mutated five residues (Gln90, Gln50, Lys93, Glu154, and Lys153) in the simulated binding site to alanine, respectively. Based on activity assays, those single mutations did not significantly affect the binding to substrate LPA, as the mutant proteins exhibited similar LPA activity to the WT protein (Fig. 6*C*). However, mutations K153A and E154A noticeably affected the binding to XY-55, that is the IC_50_ value of K153A (38.94 μM) or E154A (77.71 μM) was increased by 2- and 4-fold compared to WT (17.24 μM), respectively (Fig. 6*D*). This indicates that these sites are crucial for PgpB inhibitor binding. Consistent with the broad substrate selectivity, this finding suggests that PgpB has a large capacity to adapt to multiple ligand-binding conformations. Supporting this hypothesis, the triple mutation of K93A/K153A/E154A completely abolished PgpB activity (Fig. 6*C*). Additionally, the precise charge distribution of charged residues in the active site entry is essential for PgpB catalysis; charge-switching between Glu153 and Lys154, such as K153E or E154K, completely abolished LPA activity. These charged residues may collectively orient ligands into the large active site, which could explain PgpB’s broad substrate selectivity.

## Discussion

**Figure 7:**
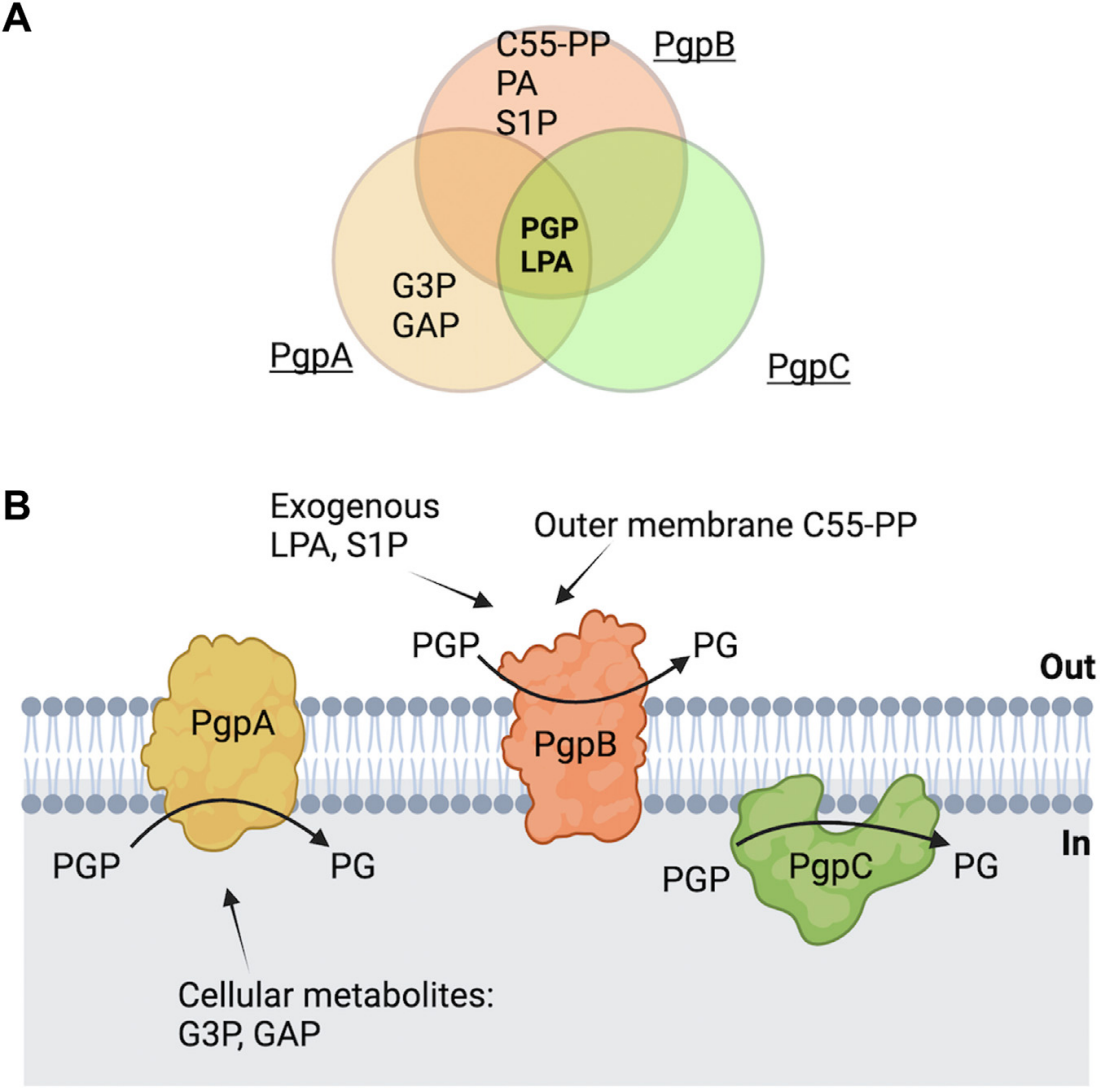
**Substrate selectivity and functional implications of three membrane phosphatases, PgpA, PgpB, and PgpC in *Escherichia coli*.***A,* substrate selectivity profiles of PgpA (*yellow circle*), PgpB (*red circle*), and PgpC (*green circle*) showing their common substrates phosphatidylglycerol phosphate (PGP) and lysophosphatidic acid (LPA). PgpA exhibits specific activities with soluble metabolites including glycerol-3-phosphate and glyceraldehyde-3-phosphate, whereas PgpB dephosphorylates a broad range of lipid phosphates including undecaprenyl pyrophosphate (C55-PP), phosphatidic acid (PA), diacylglycerol pyrophosphate (DGPP), and sphingosine-1-phosphate (S1P). *B,* hypothetical cartoon model illustrating the possible functions of PgpA, PgpB, and PgpC in the bacterial inner membrane. PgpA and PgpC catalyze Mg^2+^-dependent PGP dephosphorylation reactions to generate phosphatidylglycerol (PG) in the cytoplasmic membrane surface. PgpA may also dephosphorylate glycerol-3-phosphate, the key metabolites in bacterial glycerol and glycolytic metabolisms. On the periplasmic surface, PgpB may perform multiple dephosphorylation reactions, not only PGP but also C55-PP for peptidoglycan biosynthesis, or exogenous LPA or S1P derived from host cells.

In this study, we investigated the mechanistic and functional distinctions among the three bacterial PGP phosphatases: PgpA, PgpB, and PgpC. These enzymes play a critical role in bacterial phospholipid biosynthesis by catalyzing the dephosphorylation of PGP to form PG. Despite the identification of these three phosphatases decades ago (15, 16, 29), the reason for the presence of all three in bacterial genomes remains unclear. While each enzyme can individually facilitate the conversion of PGP to PG, our research uncovers distinct substrate preferences and ligand recognition mechanisms among them as summarized in Figure 7*A*. These differences suggest that each phosphatase may have unique or specialized functions beyond simple PGP dephosphorylation, offering new insights into bacterial lipid metabolism.

PgpB exhibits considerable versatility, adapting to a variety of lipid phosphate substrates, including LPA, PA, DGPP, S1P, and C1P (Fig. 2*D*), as well as synthetic substrate analogs XY-5, XY-14, and XY-55 (Fig. 5*B*). This broad adaptability suggests that its substrate binding pocket can accommodate multiple lipid phosphate molecules. Our ligand docking models support this idea, showing that the head groups of LPA and XY-55 adopt different binding poses, while their fatty chains are similarly stabilized in the lipid tunnel (Fig. 6, *A* and *B*). This binding conformation is consistent with our crystal structure showing that a PE lipid molecule rests its acyl chain in the lipid tunnel (18). This conserved conformation of the acyl chain might be crucial for substrate recognition, potentially aiding in orienting the phosphate headgroup toward the catalytic site. Testing with soluble metabolites indicates that substrate binding and/or catalysis depend critically on the presence of acyl chains, as the absence of an acyl chain, as seen with G3P, completely abolished catalytic activity (Fig. 4*B*). Additionally, structural analysis reveals that charged residues, Lys153 and Glu154, function as gatekeepers, positioning the phosphate headgroup in a large cavity for dephosphorylation (Fig. 6, *A* and *B*). PgpB is part of the lipid phosphate phosphatase family and shares a conserved catalytic triad with other members (30). The broad substrate versatility is a common feature of mammalian LPP homologs. For example, mammalian LPPs dephosphorylate various lipid phosphates, such as LPA, PA, S1P, and C1P, similar to PgpB (31). XY-14 inhibits both bacterial PgpB and mammalian lipid phosphate phosphatase (Fig. 5*B*). Therefore, the substrate-binding mechanism of PgpB may apply to the LPP family, which could support their multiple biological functions in lipid metabolisms.

In contrast to PgpB, PgpA demonstrates a remarkable specificity for LPA and other soluble 3-carbon sugar phosphates such as G3P (Figs. 2*C* and 4*B*). The pronounced activity of PgpA with G3P and its lack of requirement for fatty acid chains suggest a unique binding and catalytic mechanism. The kinetic analyses indicate that the substrate binding affinity (*K*_*m*_) for G3P (deacylated LPA) was modestly reduced by 4-fold from LPA (acylated G3P), while its *V*_*max*_ was increased by 2-fold (Figs. 3*A* and 4*C*). The enzyme’s active site appears adapted to recognize the glycerol moiety of its substrates, which is critical for its function. The significantly reduced activity with other 3-carbon sugar analog substrates like DHAP, GAP, and 3PG highlights the stringent requirements of PgpA’s substrate-binding site (Fig. 4*B*). Even the single fluorine substitution on the glycerol backbone of LPA, such as XY-5, abolished the PgpA activity, further supporting this notion (Fig. 5*D*). These observations point to a highly specialized role for PgpA, possibly in regulating the concentration of glycerol-derived metabolites in the cell, beyond just PGP dephosphorylation.

PgpC shares some substrate specificity with PgpA and PgpB but exhibits a distinct profile. Like PgpA, PgpC does not hydrolyze PA and shows limited activity with DGPP, which aligns with a possible steric hindrance from the sn-2 acyl group (Fig. 2*C*). However, PgpC does not act on soluble metabolites such as G3P (Fig. 4*B*), distinguishing it from PgpA and highlighting the strict dependency of an acyl chain in its substrate recognition. The inability of PgpC to process S1P and C1P further supports a substrate preference that aligns more closely with LPA and PGP (Fig. 2*C*). Taken together, PgpC may require both glycerol moiety and acyl chain for substrate recognition. This specificity might suggest a role for PgpC in processes where precise regulation of lipid phosphate levels is critical.

### Functional implications and limitations

The functional diversity observed among PgpA, PgpB, and PgpC suggests that each enzyme is adapted to fulfill distinct roles within bacterial lipid metabolism. PgpB’s broad substrate range and flexible binding mechanisms position it as a key player in managing various lipid phosphates, while PgpA’s specialized activity with glycerol-derived metabolites indicates a regulatory role in metabolic pathways. PgpC, with its selective activity, may also contribute uniquely to lipid homeostasis.

Based on these data, we propose a hypothetical model for the possible functions of three phosphatases (Fig. 7*B*). PgpB is the only lipid phosphatase identified on the periplasmic surface or outer surface of bacteria. In addition to its C55-PP activity previously identified (19), PgpB might act to detoxify or modulate the levels of LPA or S1P derived from the host. LPA/S1P are bioactive lipids present in animal tissues and biological fluids (32). Several lines of evidence suggest that host LPA/S1P attenuates bacterial infections (33, 34). These detergent-like lysolipids could potentially affect bacterial membrane integrity or signaling pathways if present in excess (35). By dephosphorylating LPA, PgpB could regulate LPA levels, thus contributing to the maintenance of membrane homeostasis and preventing potential disruptions caused by excess LPA.

On the cytoplasmic surface, PgpA’s selective dephosphorylation of G3P underscores its potential role in regulating cellular levels of this important metabolite (Fig. 7*B*). G3P is a key intermediate in glycolysis and lipid metabolism. In bacteria, its levels must be tightly regulated to support energy production and membrane formation (36). Given PgpA's ability to dephosphorylate G3P, it is plausible that PgpA may function as a regulatory checkpoint for G3P, controlling its availability for downstream metabolic pathways. By dephosphorylating excess G3P, PgpA could help maintain a balance between energy production and lipid synthesis, ensuring that G3P is not excessively directed toward phospholipid biosynthesis at the expense of glycolytic energy production. This regulatory mechanism might be particularly important under varying growth conditions or metabolic stress, where the demand for membrane lipids *versus* energy production fluctuates. Hypothetically, PgpA's enzymatic activity on G3P could play a potential role in maintaining metabolic homeostasis and adapting to physiological needs.

The functions of PgpC remain less clear. PgpC’s catalysis is only limited to LPA and PGP (Fig. 7*A*). It is uncertain if PgpC could regulate the content of endogenous LPA generated as a lipid precursor in the inner leaflet. Alternatively, PgpC may function primarily as a PGP phosphatase, compensating for deficiencies in other enzymes involved in glycerol or lipid metabolism. For instance, if PgpA or PgpB is occupied for G3P regulation or C55-PP catalysis, PgpC might compensate for their roles in PG biosynthesis. This functional overlap could be crucial for maintaining metabolic homeostasis in bacterial cells. The presence of multiple phosphatases could provide a backup mechanism to ensure that PG levels are properly maintained even if one or two enzymes are impaired, as seen in the previous mutational studies (15).

While the *in vitro* findings from this study provide valuable insights into the substrate specificity and potential functions of PgpA, PgpB, and PgpC, they come with inherent limitations that must be addressed. The characterization of these phosphatases in a controlled *in vitro* environment, although informative, does not fully replicate the complex physiological conditions of living bacterial cells. Enzyme activity and substrate interactions observed *in vitro* may differ significantly under *in vivo* conditions due to factors such as cellular compartmentalization, interactions with other biomolecules, and the influence of the cellular microenvironment. For instance, the activity of PgpB on exogenous lipids like LPA and S1P, as well as the role of PgpA in regulating G3P, must be validated in living organisms to confirm their physiological relevance. Therefore, further research involving genetic manipulation and functional assays in live bacterial systems is necessary to validate these findings and fully understand the biological significance of these phosphatases in their natural context. Nonetheless, this study lays a foundation for uncovering the functions of these membrane phosphatases in bacterial metabolism in the future.

## Experimental procedures

### Chemicals

Phospholipid substrates including 18:1 LPA (1-(9Z-octadecenoyl)-sn-glycero-3-phosphate), 16:0 PA (1,2-dihexadecanoyl-sn-glycero-3-phosphate), 16:0 S1P ((2S,3R, 4E)-2-aminooctadec-4-ene-1,3-diol-1-phosphate), 16:0 C1P (N-palmitoyl-3-deoxy-ceramide-1-phosphate), 18:1 DGPP (dioleoylglycerol pyrophosphate), and 18:1 PGP (1,2-dipalmitoryl-sn-glycero-3-[phospho-rac-(1'-(3′-phospho)glycerol)]) were purchased from Cayman or Avanti Polar Lipids. n-dodecyl-ß-maltoside (DDM) is a product of Anatrace. G3P, DL-glyceraldehyde-3-phosphate, dihydroxyacetone phosphate, 3-phosphoglyceric acid, and p-nitrophenyl phosphate were purchased from Sigma or Thermo Fisher Scientific. XY-14 was purchased from Echelon. XY-5 and XY-55 were a gift from Prof. Glenn Prestwich, the University of Utah. The molecular weight of XY-5 and XY-55 was confirmed by electrospray ionization mass spectrometry analysis in the Rice University Mass Spec Facility (Fig. S1).

### Protein expression and purification

The *pgpA*, *pgpB*, and *pgpC* genes from the *E. coli* genome were individually cloned into the pET-28a vector (Novagen), creating plasmids for the expression of N-terminal His-tagged proteins. Site-directed mutagenesis was employed to introduce specific point mutations. All plasmid constructs were verified by sequencing to ensure the accuracy of the modifications.

Protein expression was carried out in *E. coli* BL21 (DE3) cells using an autoinduction method (37). Briefly, cells harboring the expression plasmids were grown in an autoinduction medium at 37 °C for 3 h, followed by continued incubation at 18 °C for 20 h. The cells were then harvested and resuspended in lysis buffer containing 50 mM Tris–HCl (pH 8.0), 500 mM NaCl, and 10% glycerol. Cell lysis was achieved by passing the suspension through an EmulsiFlex-C3 homogenizer (Avestin) at 15,000 psi for three cycles.

Following lysis, the cell debris was removed by centrifugation, and the membrane fractions were collected by ultracentrifugation at 40,000 rpm for 1 h. The membrane pellets were solubilized in lysis buffer supplemented with 1% (w/v) DDM at 4 °C for 1.5 h. The solubilized membrane proteins were then loaded onto a nichel-charged nitrilotriacetic acid (Ni^2+^-NTA) column (GE Healthcare) pre-equilibrated with lysis buffer containing 0.05% DDM and 20 mM imidazole. After washing, proteins were eluted with a buffer containing 50 mM Tris–HCl (pH 8.0), 500 mM NaCl, 400 mM imidazole, and 0.05% DDM. Protein buffer exchange was performed using a desalting column (GE Healthcare) equilibrated with 20 mM Tris–HCl (pH 8.0), 200 mM NaCl, and 0.05% DDM. Protein concentrations were determined using the Bradford protein assay (Bio-Rad).

### Colorimetric phosphatase assay

Phosphatase activity was assessed *in vitro* using a colorimetric malachite green assay (38). In the assay, Pi generated from lipid dephosphorylation reactions reacts with malachite green molybdate under acid conditions to form a complex, which can be quantified spectroscopically. The colorimetric reagent was prepared by mixing 34 mM ammonium molybdate in 5 M hydrochloric acid with 2.16 mM malachite green oxalate in a 1:3 (v/v) ratio at room temperature, followed by filtration. All substrates were prepared in a reaction buffer composed of 20 mM Tris–HCl (pH 7.5), 200 mM NaCl, 2 mM MgCl_2_, 4% glycerol, and 0.05% DDM for immediate use.

Each assay was conducted in a 100 μl reaction mixture at room temperature, containing 0.5 μg PgpA, 0.05 μg PgpB, or 1.5 μg PgpC protein. For kinetic analysis, no more than 30% of the substrates were hydrolyzed in the reactions for 5 min. The reaction was terminated by adding 200 μl of the colorimetric reagent, followed by 30 μl of 1% polyvinyl alcohol. The mixture was then spectroscopically measured at 660 nm. The concentration of free Pi was determined based on a standard curve created using potassium phosphate.

For kinetic analysis, reactions were performed with varying substrate concentrations for 5 min. The data were analyzed using GraphPad Prism software (www.graphpad.com) and plotted according to the surface dilution model, as described by Carman *et al.* (39). For inhibitor studies, XY-14 or XY-55 was incubated with the proteins for 10 min at room temperature before adding 115 μM LPA substrate. The reaction time was 5 min. IC50 values were generated by fitting the data to single-exponential binding curves using GraphPad Prism software. All assays were conducted in triplicates as indicated.

### Molecular docking

The X-ray structure of PgpB [PDB ID: 5JWY] in complex with PE resolved at the resolution of 3.2 Å was retrieved from the Protein Data Bank (18). The PgpB structure [PDB ID: 5JWY] was preprocessed using the protein preparation wizard module in Schrödinger Maestro software (Protein Preparation Wizard, Schrödinger, LLC; https://www.schrodinger.com/platform/products/maestro/). The 3D structures of two ligands LPA and XY-55 were prepared using LigPrep (LigPrep, Schrödinger, LLC). The Glide module was used to perform the molecular docking (Glide, Schrödinger, LLC), where the Receptor Grid Generation Panel within the Glide suite was utilized for generating the receptor grid criterion— 18 Å per side for the inner cubic grid box and 30 Å per side for the outer box, with the centroid encompassing the catalytic triad region (His163, His207, and Asp211) of the protein structure and the phosphate headgroup of crystal ligand PE. The OPLS4 force field was employed for the evaluation and ranking of the ligand docking poses. For each docked compound, its best-scored docking pose was selected for further analysis and visualized using PyMOL (www.pymol.org) (40).

## Data availability

All data are contained within the article.

## Supporting information

This article contains supporting information.

## Conflict of interest

The authors declare that they have no conflicts of interest with the contents of this article.
