# Supplementary material for: Mechanistic diversity and functional roles define the substrate specificity and ligand binding of bacterial PGP phosphatases
Source: J Biol Chem. 2024 Nov 5;300(12):107959. doi: 10.1016/j.jbc.2024.107959 (PMC11629553; doi:10.1016/j.jbc.2024.107959)
Supplement: Supplemental Fig. S1 [file mmc1.pdf]

a

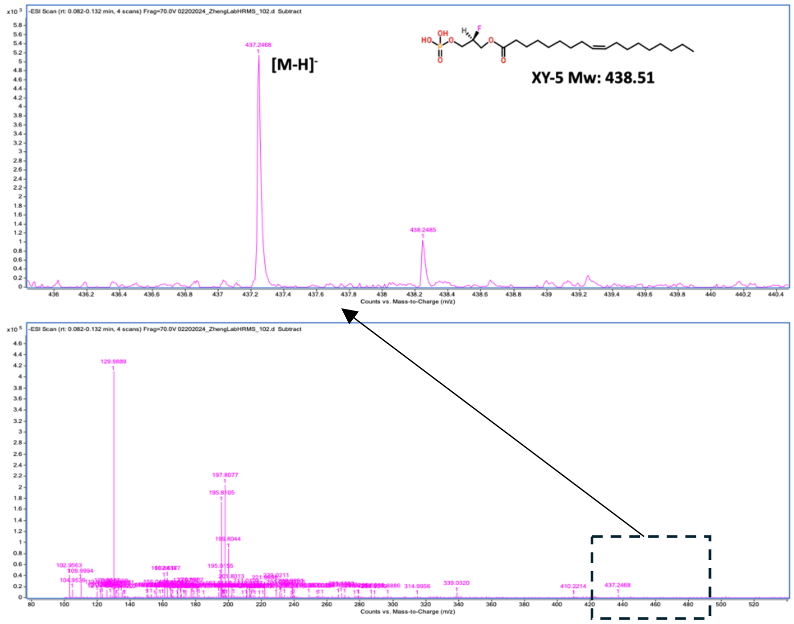

b

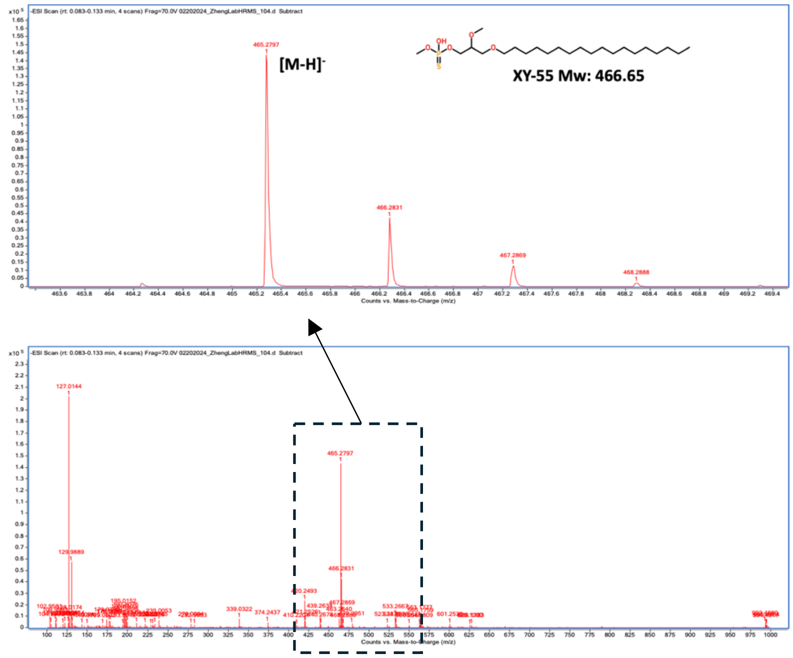

Figure S1. ESI-MS analyses of synthetic substrate analog compounds. a) XY-5; b) XY-55. Mass Spec analyses confirmed the molecular weight of these synthetic compounds. Analyses were performed in the Rice University Mass Spectroscopy Facility.
